# Enhancing User-Interviewing During Design Thinking: Leveraging Clinical Communication Skills to Augment the Student-Innovator’s Toolkit

**DOI:** 10.1007/s40670-025-02437-1

**Published:** 2025-06-04

**Authors:** Kagiso Dikgomo, Raeesah Ackerdien

**Affiliations:** Department of Medical Practice, Medical School, Faculty of Health Sciences, Nelson Mandela University, Gqeberha, South Africa

**Keywords:** Clinical communication skills, Consultation models, Design thinking, Teaching design thinking, User-interviewing

## Abstract

The pedagogical application of design thinking in higher education has increased in recent years. Many higher education institutions apply design thinking to acquaint students with user-centred approaches for problem-solving. We consider a problem identified in an evaluation of experiences in implementing design thinking in a master’s-level course, where it was discovered that student designers had limited experience in qualitative interviewing skills, which compromised the quality of user engagement. We provide a potential solution for design thinking courses to improve user interviewing. Accordingly, we make curriculum framework and faculty development suggestions. Lastly, we suggest a research agenda to coincide with this solution.

## Introduction

The application of user-centred design approaches for innovation has gained prominence in recent years, including in higher education [1]. Several universities have implemented these approaches for pedagogical purposes, ranging from undergraduate to postgraduate programmes in social science, design, art, business, and engineering disciplines [2]. In health professions education, user-centred design approaches have been applied in various disciplines, including medicine, nursing, pharmacy, physiotherapy, occupational therapy, and public health [1, 3]. The pedagogical application of user-centred design approaches is widespread, including world regions like the Americas [1], Europe [4], and Africa [5].

An example of a user-centred design approach is *design thinking*. It is a systematic process that is characterised by overlapping and iterative phases and involves the end-user throughout to develop contextually appropriate solutions [6]. McLaughlin et al. [2] highlight that for the successful implementation of design thinking, a diverse multidisciplinary team characterised by multiple perspectives and backgrounds is required. There are several influential models of design thinking [7], including the Hasso Plattner Institute of Design at Stanford University (d.school) model, which consists of five modes, namely empathise-define-ideate-prototype-test. In this paper, we comment on the phenomenon of user interviewing during the empathise mode.

To *empathise*, design thinking implementers would apply engagement techniques such as observation, user-interviewing, and immersion [8]. Doorley et al. [8] instruct that designers should adopt a “beginner’s mindset” and ask *why* questions from a point of curiosity, ask a variety of questions in a neutral manner, encourage users to tell their stories, listen attentively, and to embrace silence. The authors propose that interviews should begin with introductions, building rapport and eventually exploring or evoking stories and emotions while questioning users’ statements. These techniques are used to generate a contextual understanding of the user, their behaviour, and how they interact with others and their environment. This understanding is then used to formulate a Point of View in the *define* mode — it functions as a problem statement or challenge that guides the development of a solution. In the *ideate* mode, design thinking implementers transition to exploring a solution to the problem previously identified. During the subsequent *prototype* mode, inexpensive physical low-fidelity prototypes are generated to test functionality. These can take various forms, such as objects and role-plays. The low-fidelity prototype is then tested during the *test* mode, in which it is placed in the user’s context. The iterative nature of design thinking facilitates further learning and understanding of the user and their context, which plays a role in refining prototypes and solutions.

### Context

The application of design thinking for pedagogy in higher education has not been without problems. A recent evaluation of the implementation of design thinking in higher education [5] identified that student designers had limited experience in qualitative interviewing. An implication of this was that student designers struggled to maintain conversational flow. This in turn “compromised the quality of the engagement” (pp. 108) [5]. When the quality of user engagement is compromised, subsequent modes of the design thinking challenge may be compromised. For instance, student designers could miss critical information which can help understand the user or their environment, thus leading to a misguided Point of View. Although iteration is a part of the design thinking process, compromised quality of user engagement may become a vicious cycle of not solving the problem. This may have a negative impact on students’ learning experiences. We are of the view that clinical communication skills can offset challenges faced by students who are not trained in clinical or qualitative interviewing. Clinical communication enjoys much research supported by empirical evidence in both higher education and clinical practice. Given that design thinking emphasises diverse multidisciplinary teams and perspectives for success, it is worthwhile finding solutions to the problem identified by van der Westhuizen et al. [5].

## Clinical Communication Skills and Models

Clinicians routinely conduct consultations as part of clinical practice — these often involve collecting a medical history. A variety of communication skills are applied to facilitate this process [9]. These skills are used to facilitate the clinical interview between a healthcare practitioner and a patient.

Various models have been devised to facilitate the clinical interview. The combination of clinical interviewing models and communication skills has valuable implications for both healthcare practitioners and patients. When applied effectively, communication skills have a positive impact on patients’ health outcomes, health literacy, self-efficacy and medication adherence [10], and satisfaction [11]. Successful application of clinical interviewing models allows clinicians to problem-solve interactional problems [12]. The effective application of communication skills and clinical interview models is thus crucial in healthcare practice.

A well-researched example of a clinical interviewing model is the Calgary-Cambridge guide [9]. It integrates *content* (i.e. the biopsychosocial information of the patient’s problem), along with *process* skills (i.e. clinical communication skills) to achieve the goal of comprehensive history taking. It consists of several “tasks” which guide the clinical consultation — these include Initiating the Session, Gathering Information, the Physical Examination, Explanation and Planning, and Closing the Session. Here, we briefly outline the first two tasks as they have direct relevance to user-interviewing in design thinking.

To begin a clinical interview or consultation, i.e. Initiating the Session, a clinician would aim to establish initial rapport by greeting a patient and obtaining their name while introducing themself respectfully, attend to the patient’s physical comfort as appropriate, and show interest in the patient. The clinician would then identify the reasons for the patient consulting and negotiate a shared focus for the consultation. Prior to commencing with the Gathering Information task, a clinician would seek permission to continue. To begin this task, the clinician would first encourage the patient to “tell the story of the problem(s) from when first started to present, in [their] own words” (pp. 44) [9]. Throughout this task, the clinician would explore the patient’s problems in detail by applying various questioning and structuring techniques and attend to the therapeutic relationship.

We note that how clinicians might conduct the first two tasks of the medical consultation is similar to how Doorley et al. [8] have conceptualised user-interviewing. The operationalisation of clinical interviewing, however, appears to be more explicit, expansive, and enjoys extensive research. A reason for this is that communication skills are an integral part of clinical competence. In the Calgary-Cambridge guides, process skills for executing introductions, collecting information, building rapport, and attending to flow are delineated, unlike in the d.school design thinking toolkit. Additionally, the Calgary-Cambridge guide outlines process and perceptual skills for exploring a patient’s problem [9]. We posit that design thinking implementers could benefit from adopting elements of the Calgary-Cambridge model and related clinical communication skills. We posit that this can promote conversational flow and quality of engagement between designers and users.

## How Clinical Communication Skills Can Be Integrated in User-Interviewing

### Teaching Implications

The integration of clinical communication skills to strengthen user-interviewing in design thinking is not without pedagogical implications. To operationalise this integration, additional inputs relating to curriculum development and faculty development are required [13].

#### Curriculum Development

Existing courses that integrate design thinking would need to adapt their curricula by adding relevant learning outcomes and content and devising appropriate teaching and assessment strategies. Recent evidence from health professional education (e.g. Psychology [14], Medicine [15], and Nursing [16]) suggests that training students in communication skills, including the Calgary-Cambridge model, improves their clinical interviewing. We posit that the same would apply to the integration of clinical communication skills in user-interviewing during design thinking. In Table 1, we present an example of how existing design thinking curricula could be adapted.

**Table 1:** Example of clinical communication skills learning outcomes and teaching and assessment strategies for design thinking curricula adaptations to support user-interviewing

Learning outcomes	Teaching strategies [9, 17]	Assessment strategies [9, 18, 19]
Identify and Describe the (model) and selected clinical communication skills for the clinical interview	Didactic lectures to introduce the basic aspects of a clinical communication model and related skills and how to apply them in a design thinking context	*Assessment of knowledge* Written assessments* assessing students’ ability to identify and describe relevant communication skills and a clinical interview model
Apply the (model) and selected clinical communication skills for user-interviewing during design thinking	Skills spotting videos to illustrate and analyse the application of concepts	*Assessment of knowledge* Written assessments* assessing students’ ability to apply clinical communication skills and a clinical interview model by analysing and synthesising a user-interview dialogue
Simulate a user-interview applying relevant aspects of the (model) and skills for effective communication	Role-playing and simulating a user-interview to practice application of clinical communication skills as they relate to design thinking, in small groups with a trained facilitator and simulated user	*Assessment of competence* Demonstration of competence in effective user-interviewing in design thinking, applying appropriate clinical communication skills alongside a clinical interview model *Other forms of assessment* Written reflections^¶^

*Written assessments may take the form of single best answer tests, short answer and long/case question tests, etc.

^¶^Written reflections must be based on the experience of learning and applying communication skills (practice) and may be prepared in line with short-form, e.g. Fogarty [20] or traditional/longer-form, e.g. Gibbs [21] approaches

#### Faculty Development

Although there are instances when design thinking is applied with clinically trained instructors (e.g. van de Grift & Kroeze [4]), in some instances, this may not be the case. As such, not all course instructors may be familiar with concepts in clinical interviewing and relevant teaching and assessment methods. Therefore, faculty development would be required for effective uptake and implementation. This is specifically to train instructors in clinical communication skills, models, and how to teach (e.g. use of various modalities like skills spotting and simulation) and assess clinical interviewing (albeit in a design thinking context). Collaboration with clinical skills training units for delivery of new teaching content, especially in practical aspects, may be of value. Sandars and Goh [13] suggest that such collaborations are important for faculty development.

### How might students apply clinical communication skills for user-interviewing?

To illustrate how students can apply elements of the Calgary-Cambridge model and selected clinical communication skills, we provide sample statements and questions, and established communication techniques and frameworks in Table 2. These examples are aligned with the user-interviewing approach outlined in the d.school toolkit — here, we align the integration of clinical communication skills in user-interviewing to the following tasks in the Calgary-Cambridge model: Initiating the Session, Gathering Information, and Closing the Session.

**Table 2:** Conversational overview of how student designers can apply clinical communication skills and a model for user-interviewing

Tasks in Calgary-Cambridge model [9]	Interviewing for empathy in d.school method [8]	Techniques, statements, and questions that design thinking implementers can apply from clinical communication skills
Initiating the Session	Introduce yourself	The d.school toolkit recommends introducing oneself to start the interview. However, it does not recommend *how* this might be achieved. We suggest the following dialogue as an exemplar, to *meet and greet* the user [22]: “Hello, my name is [full name].” A preferred name can also be suggested. Request user’s name Follow-up: “What name would you like me to use?”
	Introduce project	Similar to introducing oneself, the d.school toolkit does not recommend how this might be achieved. We suggest the following dialogue as an exemplar to introduce the project, *role*, and interview: “I am a student at [state course, department, and university name in full]. I am part of a group of students tasked with… This may involve…” “I would like to talk to you about this today if that is okay.” *(gaining consent)*
	Build rapport	Similar to introducing oneself and project, the d.school toolkit does not recommend how this might be achieved. In addition to our exemplars above, several other techniques can aid *establishing initial rapport*: • It would be useful for student designers to smile comfortably, attend to the user’s comfort, and apply the SOLER* technique (or similar) for *non-verbal communication* [23] • A *confidentiality statement* may be included, for instance; “everything we discuss will only be known to the student team and our course facilitators.” • To create a space for reciprocal dialogue, student designers may also say, “if you have any questions, you may ask at any point.” • Demonstrating respect and interest towards the user • To transition from the “meet-and-greet”, student designers may employ *structuring statements*. For instance, “our discussion may take about [time estimate], is that okay? I’d also like your permission to take some notes.”
Gathering Information	Evoke stories and question statements	To evoke stories, the d.school toolkit recommends asking questions neutrally, avoiding leading questions, and asking what-how-why questions. We suggest the addition of the following techniques for effective interviewing: • To begin evoking stories and questioning statements, student designers can apply the *patient’s narrative*. This would allow student designers to capture the users’ views of when the problem may have begun • Cone questioning technique [24], which begins with an open question, then moves to a focussed open question, and finally on to a closed question. This technique would allow the user to first describe issues in their words, then allowing the student designer to *clarify* details with closed questions • Core skills for Motivational Interviewing [25]. These skills would allow the student designer to question issues with a repertoire of skills which demonstrate interest, listening, and empathy. The skills entail: ○ Asking *open questions* to invite a user to tell their story in their own words ○ Using *affirmations* to show positive regard ○ Using *reflective listening* to express empathy ○ Using various ways of *summarising* throughout the interview, for example, an end-of-discussion summary • Student designers can also integrate the *patient’s perspective* of the Calgary-Cambridge model [9] to systematically explore and understand users’ personal experiences of a problem. This entails exploring the following framework: ○ *Ideas of cause*, for example, “what do you think is causing…” ○ *Concerns*, for example, “what are you worried about regarding…” ○ *Expectations*, for example, “how do you think we, as students trying to solve this issue, could help you?” ○ *Feelings*, for example, “how does…make you feel?” ○ *Effect on life*, for example, “how does…affect daily activities?”
Gathering Information; throughout interview	Explore emotions	The d.school toolkit recommends that interviewers must pay attention to non-verbal cues and explore emotions. It also recommends that interviewers must embrace silence. To facilitate exploration and responding to emotions more specifically, we suggest that student designers could apply the following four-step process to *respond empathically to emotions* [26]. This process would equip student designers to respond appropriately to emotion: 1. Looking out for or seeking emotion (this may be direct or indirect by inquiring about “impact on life”) 2. Identifying emotion and naming it. For example, “you said…frustrated you?” 3. Exploring reason/s for the emotion by asking open-ended question/s. For instance, “now, you mentioned that you are concerned about…please tell me more.” 4. Validating emotions by expressing an empathic/validating response. For instance, “you were perfectly correct to think that way.”
Closing the Session	Thank and wrap-up	The d.school toolkit recommends that interviewers must thank their user as part of wrapping-up the interview. However, it does not outline how this might be achieved. We suggest the following techniques: • *Summarising* as described above, to check that there is mutual understanding of the information collected • Use of a *structuring statement*. For example, “thank you for sharing your experience of…do you have any questions for me at all? We have come to the end of our discussion, going forward…”

*SOLER, sitting square, open posture, leaning slightly forward, eye contact, relaxed posture

## Implications for Future Research

In our view, the integration of clinical communication skills in design thinking is multifaceted, with curriculum, teaching, learning, assessment, implementation, and evaluation implications. To understand and develop the different elements herein, educational programmes would benefit from conducting context-specific research alongside any adaptations to design thinking curricula. This research may also seek to integrate appropriate implementation theories, models, and frameworks (see Nilsen [27]) to illustrate planning, implementation, and evaluation processes and outcomes. For instance, courses integrating clinical communication skills may apply process models such as the Knowledge-to-Action Framework [28] to outline how evidence was translated into practice. Qualitative investigations on faculty development and learning and teaching may be included to elucidate experiences and practice recommendations. To determine the success of the integration of clinical communication skills for user-interviewing in design thinking, implementation outcomes can be selected from Proctor et al.’s [29] taxonomy of implementation outcomes for investigation. For instance, courses may assess programme *acceptability* among faculty and students. To generate an understanding of influences on outcomes, theories such as the Normalisation Process Theory [30] can be employed. Additionally, course instructors may assess the effectiveness of their teaching approaches on students’ learning using various quasi-experimental designs (see Siedlecki [31]) such as the *One-Group Pretest–Posttest* design. To do this, course instructors should develop and assess the reliability of appropriate competency checklists. The overarching goal of such research activities would be to determine whether training faculty and students in relevant clinical communication skills and models improves user-interviewing during design thinking. Educational research of this nature is especially important, since the pedagogical application of design thinking in higher education is still relatively new.

## Conclusion

The integration of clinical communication skills during user-interviewing in design thinking carries several implications, including the recurriculation of design thinking courses, faculty development, and scholarship of teaching and learning. We believe that such an integration may benefit educational programmes which apply design thinking for innovative problem-solving, particularly those programmes outside of the health professions. Design thinking courses that wish to integrate clinical communication skills for user-interviewing may benefit from collaboration with established clinical skills training units which can support planning, faculty development, implementation, and programme evaluations. Enhancing student designers’ user-interviewing skills can promote quality of user engagement, thus addressing the problem identified by van der Westhuizen [5].
